# >Modern Contraception: Uptake and Correlates among Women of Reproductive Age-Group in a Rural Community of Osun State, Nigeria

**DOI:** 10.4314/ejhs.v30i4.8

**Published:** 2020-07-01

**Authors:** Ajibola Idowu, Grace Chinyere Ukandu, Jeremiah Mattu, Damilola Olawuyi, Adeola Abiodun, Phillip Adegboye, Chiamaka Chibu-Jonah, Anita Eseogene Siakpere, Anita Eseogene Ishola, Titilola Adeyeye, Samuel Alabi

**Affiliations:** 1 Department of Community Medicine, Bowen University, Iwo, Nigeria

**Keywords:** Contraception, family planning, knowledge, attitude, practice, uptake, Nigeria

## Abstract

**Background:**

Universal contraceptive access is one of the key strategies for achieving sustainable developments in any country. Yet, uptake has remained low in most developing nations like Nigeria. The reasons for low use must be contextually understood to aid effective contraceptive programming. This study assessed contraceptive use and its determinants among reproductive aged women in Ejigbo, Osun State, Nigeria.

**Methods:**

A quantitative study involving 405 participants which were recruited using multi-stage sampling method was carried out. Data were collected using pretested semi-structured, interviewer-administered questionnaire. Chi-Square test and binary logistic regression analysis were used for inferential statistics.

**Result:**

The mean age of the respondents was 28±6. The majority (92.8%) of the respondents were aware of family planning, 68.9% of them possessed good knowledge but only 53% of them demonstrated favorable contraceptive attitude. Less than half (33.0%) of those who had heard about contraception were current users of modern methods. Injectables (45.0%) and male condoms (30.0%) were the most prevalent contraceptive methods among the respondents. The main determinants of contraceptive uptake were respondents’ educational status (AOR=0.525, 95%CI=0.284-0.972), contraceptive knowledge (OR=0.512, 95%CI=1.242-1.968) and attitude (OR=0.512, 95%CI=1.2421.968). Fear of perceived side effects (45.2%), low pregnancy risk perception (35.7%) and spousal refusal (12.5%) were the main reasons for non-contraceptive use among non-users.

**Conclusion:**

Contraceptive demand in the study population was low in spite of high awareness level. There is a need to increase contraceptive literacy in the study population and make the services more acceptable to rural dwellers so as to meet the SDG-3 target in Nigeria.

## Introduction

Voluntary control of fertility has become of paramount importance to every facet of modern society particularly due to its ability to slow down population growth and indirectly improve national economic growth. The world has continued to witness exponential growth of its population as it has been projected to reach 9.8 billion people by 2050 (1). Most of these growths have been occurring in the developing countries which have minimal resources to match, thereby perpetrating the circle of poverty in those countries. Among the most populous countries in the world, Nigeria is growing at the fasted rate with a current population of about 200 million people (ranking 7^th^ in the world) (1). Meanwhile, contraception is a key strategy of controlling this upward trend in population growth by helping to prevent unwanted and unplanned pregnancies. Asides this, adequate contraceptive utilization has the potential to reduce the rate of unwanted/unintended pregnancies and unsafe abortion. This is of paramount importance particularly in rural areas where women are mostly vulnerable to adverse pregnancy and delivery outcomes due to poor quality of maternal healthcare services available to them.

Contraceptive access is intricately related to its utilization. Thus, the United Nations has recommended that nations should ensure universal access to sexual and reproductive health care services including family planning information and education in order to achieve target-7 of the Sustainable Development Goal (SDG)-3 (2). Contraceptive access can be described in terms of its physical access, availability, acceptability, affordability and accommodativeness. It is also recommended that nations should ensure integration of reproductive health services into national developmental strategies and programs (3). Family planning service has indirect benefits of reducing maternal mortality rate (MMR) in a country by eliminating unsafe abortions which often result from unintended pregnancies.

Every year, over 40,000 women in Nigeria die from childbirths and pregnancy related complications (4). Thus, while Nigeria is responsible for only 2.5% of the world's population, it accounts for 14% of global maternal deaths every year (3). There has not been significant reduction in MMR in Nigeria considering the National Demographic and Health Survey (NDHS) reports from 2008-2018 (545,576 and 512 deaths per 100,000 live births) (4–6). This is deviant to SDG 3.1 target of reducing global MMR to as low as 70 deaths per 100,000 livebirths (2).

Improving contraceptive uptake among women of reproductive age in Nigeria is thus critical for reversing this situation. However, the 2018 NDHS reports that only 12% of Nigerian women currently use a modern form of contraception resulting in high rates of unwanted/unplanned pregnancies, unsafe abortions (6). In 2012, the rate of unintended pregnancies in Nigeria was estimated at 59/1,000 of women aged 15–49 by Bakole et al. The study also estimated that about 1.25 million induced abortion were recorded within the same year (7).

Studies have revealed variations in the types of contraception preferred by Nigerian women and their determinants (6,8). Most of the studies have focused on residents of urban communities with a few of them targeting rural dwellers (8–10). Meanwhile, uptake of family planning services has been low for rural dwellers compared to their urban communities possibly due to disparity in their socio-cultural and socioeconomic characteristics (6,11) . Moreover, there has been rural-urban differential in the demand for modern contraception as revealed by the 2018 NDHS, thus creating conspicuous knowledge gap as to the factors which may be responsible for this observed variation.

The rural-urban disparity in access to contraception is a global phenomenon which is part of the reasons for the launch of the United Nations’ FP2020 programme with the aim of reaching at least 20,000 underserved people with contraceptive services by year 2020(12).There is currently paucity of information on demand-related barriers to modern contraceptive use in most Nigerian rural communities. Thus, the current study sought to bridge this gap in knowledge and provide useful baseline information for policy makers in the field of reproductive health, to develop cost-effective family planning interventions to increase its uptake among the rural dwellers. The study assessed contraceptive knowledge, attitude and determinants of women in reproductive age group in Ejigbo, Osun State, Nigeria.

## Materials and Methods

This study which employed cross-sectional design was conducted among women of reproductive age group in Ejigbo community, the administrative capital of Ejigbo Local Government Area (LGA), Osun State, Nigeria. The population of the LGA is estimated to be 150,610 (13), comprising predominantly of farmers and people of Yoruba ethnic group. The predominant religions in the community are Islam, Christianity and Traditional. The community has four (4) accredited healthcare facilities for provision of family planning services at least once each week. Well-trained healthcare personnel such as Nurses and Community Health Officers (CHOs) are available in those facilities to attend to clients. However, there are many chemists’ shops where some family planning methods (such as male condom and emergency contraceptive pills) are obtained over-the counter. Occasionally, outreach programmes where condoms are distributed to participants occur in the community.

All women between 15–49 years of age, who were permanent residents of Ejigbo community and who consented, were allowed to participate in the study. Eligible women adjudged to have health challenges which could prevent them from giving valid responses to our questions were exempted from the study.

Our sample size was calculated using Leslie Kish formula for estimating sample size in a population which is greater than 10,000 people. The standard normal deviate was set at 1.96, which corresponds to 95% confidence level. We assumed that 25.4% of our study participants would be using modern contraceptives based on report from a study conducted in Oyo State, Nigeria, in 2017 by Adeyemi et al(14). The margin of error was set at 5%, and 10% non-Ajibola I. et al.

response rate was envisaged and correction for this was made. The sample size was multiplied by

1.2 to correct for cluster effect, and a total of 420 questionnaires were administered to increase the representativeness of our study.

Multistage sampling method was used in recruiting our eligible respondents. Firstly, two out of eleven electoral wards in Ejigbo LGA were selected by simple random method (balloting). Secondly, two Enumeration Areas (EAs), each from the chosen electoral wards, were selected by simple random technique Next, two streets each were selected via balloting from the two selected EAs. All the households on a street with eligible respondents were visited for interview. In a household with more than one eligible respondent, one was selected by simple random method.

Data were collected using interviewer-administered, semi-structured questionnaire which was developed after reviewing previous similar studies. The instrument collected information on respondents’ socio-demographic characteristics, their contraceptive knowledge, attitude, and uptake as well as barriers to modern contraception. The questionnaire was written in simple English for easy understanding. Translation into Yoruba language was done for respondents who preferred answering our questions in their native language, back-translation into English language was done to preserve original meanings of the questions. Data were collected by a group of 10 medical students who were trained on questionnaire administration in rural communities.

A pretest exercise was carried out in a different population -among 40 women of reproductive age group in Ife-Odan, using convenience sampling method. The process assisted us in strengthening the internal validity of our instrument. Irrelevant or ambiguous questions noticed were either rephrased or entirely removed in line with our study objectives.

There was daily editing of each questionnaire before data entry into Statistical Package for Social Sciences (IBM SPSS 21.0) for analysis. Data were presented in tables and charts. Mean and standard deviation were used to summarize numerical data while Chi-Square test was used to compare categorical variables. Binary logistic regression was used to examine factors that were significantly associated with current contraceptive use among the respondents. Included variables in the regression model were those which were either statistically significant at bivariate level or had been reported as strong determinants of contraceptive uptake. A 95% confidence interval was estimated for each variable to determine whether they were significant predictors of modern family planning use.

Awareness of respondents on contraception was assessed by asking if they had ever heard of modern contraception, and if they could correctly mention at least two contraceptive methods. However, knowledge on contraception was assessed using a scoring system. To do this, eight questions were asked; correct response to each of the questions attracted 1-point while wrong answers attracted 0-point. The mean knowledge score and standard deviation (SD) was calculated and was found to be 5±1.5. Those with the mean score of 3.5 or less were categorized as having poor knowledge, those with score between 3.6 to

6.5 were classified as having fair knowledge while those with at least 6.6 were grouped as having good knowledge.

Attitude of respondents to contraception was assessed using a 5-point Likert-scale ranging from strongly agreed (5 points) to strongly disagreed (1 point). In doing this, 10 questions were positively phrased on contraception. Th maximum score obtainable was 50 points and the median score (50^th^ percentile) was estimated. The median value was 27 and the range was 30 (45–15). Respondents who scored below the 50^th^ percentile were classified as having negative attitude while those who scored above the median value were grouped as people with positive attitude to contraception.

Ethical approval to conduct the study was sought from the Ethical Review Committee of Bowen University Teaching Hospital, Ogbomoso, Nigeria. Permission was also obtained from the community head and his cabinet members before commencing the study. Written consents were obtained from each respondent before they were allowed to participate in the study. Participation in the study was entirely voluntary, and participants were allowed to opt out at any stage of the interview as they so wished. Responses from the interviewees were kept strictly confidential as the questionnaires were made anonymous and data collected were entered into passworded computers.

## Results

A total of 420 questionnaires were administered, but 405 of them were returned satisfactorily completed (response rate of 96.4%). The mean age of the respondents was 28±6. Most (38.8%) of the respondents were 20-29 years of age, 64.2% of them were married and 52.1% had secondary education. Most (60.2%) of the respondents practiced Islamic religion. Most (63.0%) of the respondents had 1-2 children, 26.0% had more than 4 children and the mean number of children was 1.2±0.8. More than half (55.3%) of the respondents earned less than 30,000.00 Naira ($81.74) monthly which is the current national minimum wage in Nigeria (Table 1).

**Table 1:** Sociodemographic Characteristics of Respondents

Variable	Frequency N=405	Percent (%)
**Age (Years)**		
≤19	91	22.4
20-29	157	38.8
30-39	97	24.0
≥40	60	14.8
**Marital status**		
Single	138	34.1
Married	260	64.2
Divorced/Separated	0	0.0
Widowed	7	1.7
**Level of education**		
No formal education	70	17.3
Primary	63	15.6
Secondary	211	52.1
Tertiary	61	15.1
**Religion**
Christianity	160	39.5
Islam	244	60.2
Traditional	1	0.2
**Parity**		
None	75	18.5
1-2	255	63.0
3–4	49	12.1
>4	26	6.4
Mean	1.2±0.8	
**Average family monthly income (Naira)**		
≥30,000.00	224	55.3
31,000.00–60,000.00	120	29.6
61,000.00–90,000.00	47	11.6
≥91,000.00	14	3.5

From Table 2, 92.8% of the respondents were aware of family planning by correctly mentioning at least two contraceptive methods, most (47.0%) obtained their information from hospital staff. While 49.1% of them knew both men and women could use family planning, 44.1% stated that only women could benefit from family planning services. Almost three-quarters (71.2%) of the respondents knew there could be side effects of using family planning methods. Whereas 68.6% of the respondents possessed good knowledge of family planning, only 54.3% of them demonstrated favorable attitude to using family planning.

**Table 2:** Respondents' Knowledge Attitude and Practice of Modern Contraception

VARIABLE	FREQUENCY	PERCENT (%)
	N = 405	
**Awareness of family planning**		
Yes	376	92.8
No	29	7.2
**Sources of information**		
Hospital staff	178	47.0
Mass media	115	30.3
Family& Friends	80	21.1
Others	6	1.6
**Knowledge on who could receive family planning**	**n=376**	
Men only	23	6.2
Women only	164	44.1
Both men and women	189	49.7
**Knew if there are side effects of family planning**	**n=376**	
Yes	269	71.2
No	67	18.0
Don’t know	40	10.8
**Overall knowledge on family planning**	**N=376**	
Poor	88	23.4
Fair	30	8.0
Good	258	68.6
**Attitude of respondents to family planning**	**N=376**	
Positive	204	54.3
Negative	172	45.7
**Ever use contraception**	N=376	
Yes	160	42.5
No	216	57.5
**Current contraceptive use**		
Yes	133	33.0
No	272	67.0
**Barriers to contraceptive use among non-users**	**N=272**	
Fear of perceived side effects	123	45.2
Perceived low pregnancy risk	97	35.7
Spousal disapproval	34	12.5
Had enough children already	4	1.5
Religious factor	4	1.5
Others	10	3.6

In all, 42.5% of the respondents had ever used contraceptives but only 33.0% of them were current users. Among non-users, the most commonly mentioned barrier to contraceptive use were fear of perceived side effects (45.2%), low pregnancy risk perception (35.7%) and spousal refusal (12.5%).

**Figure 1:**
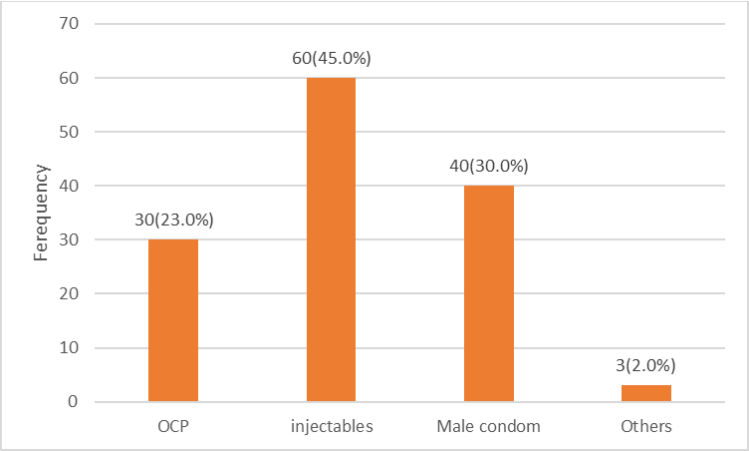
Types of modern contraceptive methods used by respondents

**Figure 2:**
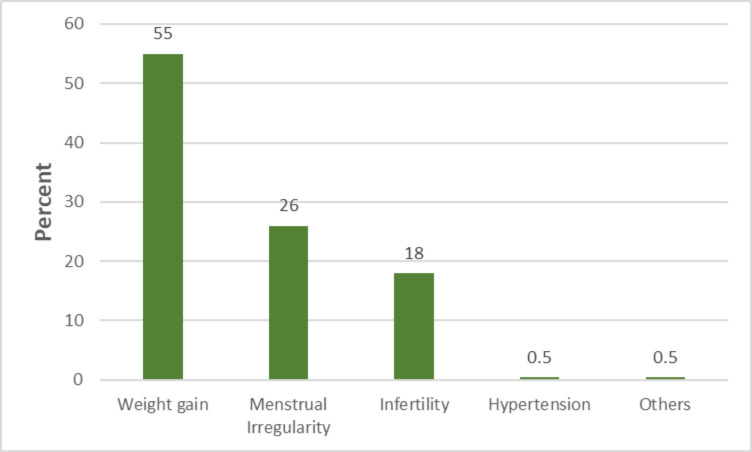
Side effects of family planning identified by contraceptive us

Injectables and male condoms were the most prevalent contraceptive methods among the respondents. They were used by 45.0% and 30.0% of the current contraceptive users respectively (Figure 1). The most commonly identified side effect of contraception was weight gain which was mentioned by 55.0% of the current users (Figure 2). In Table 3, 65.0% of women with more than four children were non-contraceptive users. Respondents with no formal education were significantly less likely to have used contraception compared to their educated counterparts (AOR=0.525, 95%CI=0.284–0.972). Likewise, those with poor knowledge and negative attitude had significantly lesser odds of modern contraceptive use (OR=0.512, 95%CI=1.242–1.968 and OR=0.158, 95%CI=0.261–0.874).

**Table 3:** Determinants of Current Contraceptive Use among the Respondents

Variable	Currently on Family planning		Total	χ^2^	*P value*	*AOR*	*95%CI*
	Yes	No					
	n=133 (%)	n=272 (%)					
**Age (Years)**				3.361	0.067	0.674	0.442 – 1.029
≤29	73(29.4)	175(70.6)	248				
≥30	60(38.2)	97(61.8)	157
**Number of living children**							
0–4	124(37.5)	207(62.5)	331	18.057	0.066	1.501	0.672–3.4110
>4	9(8.1%)	65(91.9)	74				
**Marital status**				3.617	0.057	0.650	0.416 – 1.015
Married	39(26.9)	106(73.1)	145				
Others	94(36.2)	166(63.8)	260				
**Level of education**				4.306	0.038*	0.525	0.284 – 0.972
Uneducated	15(22.1)	53(77.9)	68				
Educated	118(35.0)	219(65.0)	337				
**Monthly Income**				0.014	0.905	0.975	0.643 – 1.479
≤30,000.00	73(32.6)	151(67.4)	224				
> 31,000.00	60(33.1)	121(66.9)	181				
**Knowledge on** **contraception**				7.032	0.001*	0.512	1.242–1.968
Poor	30(25.4)	88(74.6)	118				
Good	103(39.5)	158(60.5)	261				
**Attitude to** **contraception**				3.88	0.023*	0.158	0.261–0.874
Negative	26(15.0)	149(85.0)	175				
Positive	107(53.0)	97(47.0)	204				

* Statistically significant at p=0.05, AOR=Adjusted odds ratio, CI=Confidence interval

## Discussion

The current study assessed knowledge, attitude and determinants of family planning use in a rural community of Nigeria. Almost all of the respondents were aware of family planning. High awareness level is expected to be associated with good knowledge of modern contraception, but this was not the case in the current study as 68.6% of the respondents had good knowledge. Slightly above half of the respondents had positive attitude towards modern contraception despite high awareness level. This is not unexpected since awareness has been shown to be a poor predictor of the behavioural domain of attitude (15). Thirty-three percent of the respondents were current contraceptive users. Our finding agrees with previous studies. For instance, a study by Adeyemi et al., in Ogbomoso, Nigeria, in 2016, revealed that all of the respondents were aware of contraception, but only 49.7% had ever used a method while 25.4% were current users (14). In another study conducted among Nigerian women attending antenatal clinic in Jos by Utoo et al., awareness level was 88.1%, but only 44.0% were modern contraceptive users (16). However, the modern contraceptive prevalence rate (mCPR) in the current study was much higher than the 12.0% reported by the 2018 NDHS (6) and 13.0% mCPR reported by Monjok et al in 2010 (17). The reason for the higher mCPR in the current study could have been due to a relatively high literacy rate of the respondents as 67.2% of them had at least a secondary education. However, the low mCPR recorded in the current study and other Nigerian studies is of public health importance since it could have been responsible for the high rate of unintended pregnancies and unsafe abortions in the country as reported by Bakole et al (7).

Respondents’ educational status, contraceptive knowledge and attitude were the main variables that were significantly associated with contraceptive uptake in the current study. A similar study conducted by Kasa et al., among women in resource limited area of Ethiopia corroborates our findings (18). Also, findings from the study conducted by Mitkari et al. (19), as well as that conducted by Adeyemi et al. (14), are in keeping with our results. Hence, there is a need to improve contraceptive literacy in rural communities of Nigeria. This will involve an increased and sustained political will by the Nigerian government and its partner agencies to invest more on family planning education programmes to improve the knowledge-base of rural women regarding contraception. Such programmes should also be designed using all media of communication, all-inclusive and non-discriminative strategies-to reach the underserved women and using the local languages of the rural dwellers.

As revealed in the current study, fear of perceived side effects, spousal refusal and low pregnancy risk perception were the main barriers to contraceptive use among the non-users in the study population. This findings are in congruence with the reports from previous studies (10,20–22). Contraceptive educational programme should equally target men in the rural communities for grater acceptability. Moreover, contraceptive messages will be more impactful in removing myths and misconceptions about pregnancies and family planning use if its firmly integrated in the antenatal care services.

This study may not be totally free from social-desirability bias since most of the respondents knew the interviewers were medical students. Answers might have been given to satisfy the desire of the students. However, this bias was minimized by carefully explaining the objectives of the study to the respondents and by letting them know that their honest responses to questions were necessary for actualization of the aim of the study.

In conclusion, this study reveals a high contraceptive awareness level but low knowledge, attitude and practice. The main determinants of contraceptive uptake were knowledge, attitude and respondents’ educational status. There is a need for a paradigm shift in family planning programming and delivery to rural Nigerians in order to attain the reproductive health target of SDG-3.
